# 长效重组凝血因子Ⅸ药物治疗血友病B研究进展

**DOI:** 10.3760/cma.j.issn.0253-2727.2022.03.014

**Published:** 2022-03

**Authors:** 夕妍 王, 仁池 杨

**Affiliations:** 中国医学科学院血液病医院（中国医学科学院血液学研究所），实验血液学国家重点实验室，国家血液系统疾病临床医学研究中心，细胞生态海河实验室，天津市血液病基因治疗研究重点实验室，中国医学科学院血液病基因治疗重点实验室，天津 300020 State Key Laboratory of Experimental Hematology, National Clinical Research Center for Blood Diseases, Haihe Laboratory of Cell Ecosystem, Institute of Hematology & Blood Diseases Hospital, Chinese Academy of Medical Sciences & Peking Union Medical College, Tianjin Key Laborator of Gene Therapy for Blood Diseases, CAMS Key Laboratory of Gene Therapy for Blood Diseases, Tianjin 300020, China

血友病B是一种以凝血因子Ⅸ（FⅨ）缺乏为特征的X染色体连锁的隐性遗传性出血性疾病，主要表现为关节、肌肉及深部组织自发和创伤性出血。血友病B的主要治疗方法是FⅨ替代治疗，首选基因重组FⅨ（rFⅨ）或病毒灭活的血源性凝血酶原复合物（PCC），无上述条件时可选用新鲜冰冻血浆等[1]–[2]。尽管目前应用的FⅨ制剂具有良好的疗效和安全性，但由于FⅨ半衰期较短，需要每周注射2～3次以维持保护性浓度，会造成静脉通路问题，同时加重患者经济负担，严重影响患者的依从性和治疗结局[3]。因此，临床上对于改进血友病B的治疗有着迫切的需求，即需要更长的半衰期和更优化的药代动力学特征药物以进一步改进血友病B的治疗模式。

近年来，血友病B的治疗出现了新进展。针对组织因子途径抑制物的人源化单克隆抗体Concizumab和抗凝血酶Ⅲ途径的小干扰RNA Fitusiran在血友病B临床试验中取得了良好的疗效和耐受性，基因治疗更是为血友病B的治愈带来了希望[4]–[5]。此外，蛋白工程学的进展同样为血友病B带来了潜在的创新疗法，长效rFⅨ的出现正逐渐取代过去的传统替代治疗，有望改善目前血友病B的治疗模式。长效rFⅨ半衰期显著延长，其潜在优势包括减少注射频率、提供对出血的长期保护、增加治疗依从性和改善临床结局。目前的长效rFⅨ产品包括：①聚乙二醇修饰rFⅨ：Nonacog beta pegol（GlycoPEGylated, N9-GP）；②rFⅨ-白蛋白融合蛋白（rⅨ-FP）：Albutrepenonacog alfa；③rFⅨ-Fc融合蛋白（rFⅨFc）：Eftrenonacog alfa[6]。本文对已在全球上市的长效rFⅨ药物特征和临床试验结果进行综述。三种已上市长效FⅨ基本信息见表1。

**表1:** 新型长效rFⅨ药物基本信息

药物名称	商品名	结构特征	半衰期（h）	获批适应证	公司	批准上市地区
Nonacog beta pegol（N9-GP）	Refixia^®^/Rebinyn^®^	聚乙二醇修饰rFⅨ	93.0	美国：成人和儿童血友病B按需治疗和控制出血事件；围手术期出血管理 欧洲：12岁及以上血友病B患者出血的治疗和预防	Novo Nordisk	美国（仅按需治疗）、欧洲等
Albutrepenonacog alfa（rⅨ-FP）	Idelvion^®^	rFⅨ白蛋白融合蛋白	101.7	成人和儿童血友病B：按需治疗和控制出血事件；围手术期出血管理；常规预防以降低出血发作频率	CSL Behring	美国、欧洲、加拿大、日本等
Eftrenonacog alfa（rFⅨFc）	AlprolⅨ^®^赛玖凝^®^	rFⅨ-Fc融合蛋白	82.1	成人和儿童血友病B：按需治疗和控制出血事件；围手术期出血管理；常规预防以降低出血发作频率	Biogen, Inc. and Sobi, Sanofi	美国、欧洲、加拿大、日本、澳大利亚、新西兰、中国等

注：rFⅨ：基因重组凝血因子Ⅸ；rⅨ-FP：rFⅨ-白蛋白融合蛋白；N9-GP：聚乙二醇修饰rFⅨ；rFⅨFc：rFⅨ-Fc融合蛋白

一、聚乙二醇修饰重组FⅨ：Nonacog beta pegol（N9-GP）

Nonacog beta pegol（N9-GP）是一种静脉给药的糖基聚乙二醇（PEG）化长效rFⅨ，PEG化工艺在血友病A及其他治疗领域已被证明安全有效。PEG聚合物可提高FⅨ分子的稳定性，并且可能通过减少与之连接的生物制品在蛋白水解酶、清除型受体和免疫效应细胞中的暴露，显著改善药物药代动力学（PK）属性，达到延长半衰期的目的[3]。N9-GP已被欧盟批准用于≥12岁血友病B患者预防和控制出血事件以及围术期管理，被美国食品药品监管局（FDA）批准用于儿童和成人血友病B患者的按需治疗、出血发作控制和围术期管理。N9-GP尚未在中国上市。

N9-GP的临床试验包含了Paradigm 1-4研究，主要研究对象为先前接受过治疗的成年男性血友病B患者（FⅨ活性≤2 IU/dl）。

Ⅰ期开放标签的剂量递增试验（Paradigm 1）旨在评估单剂N9-GP（剂量分别为25、50、100 U/kg）的安全性和PK。研究纳入了16例患者，除去1例因出现过敏症状退出试验，所有患者未产生FⅨ抑制物或中和抗体。相比于rFⅨ、血源性FⅨ（pdFⅨ），N9-GP的半衰期（93 h）延长了5倍，回收率分别增加了94％、20％，AUC（药时曲线下面积）分别增加了8倍和10倍；血浆清除率（CL）较rFⅨ延缓了10倍[7]。

N9-GP随机、单盲的Ⅲ期研究（Paradigm 2）旨在评估N9-GP安全性和疗效。研究纳入了74例13～70岁患者，一组接受为期52周的预防治疗（随机分为每周1次10 IU/kg或40 IU/kg剂量组），另一组接受为期28周的按需治疗。共67例患者完成研究，均未产生抑制物，未发生与N9-GP相关的严重不良事件。预防治疗疗效如表2所示[8]。另一个开放标签、单盲的Ⅲ期临床试验Paradigm 3旨在评估N9-GP在围术期止血的疗效。研究纳入了13例13～70岁计划接受重大手术的患者。所有患者术前接受了N9-GP 80 IU/kg治疗，术中止血效果均被评级为“优”（10/13）或“良”（3/13），药物安全性良好。术前单剂量N9-GP 80 IU/kg在整个手术过程中可维持FⅨ活性于所需水平，并且围术期N9-GP的消耗量和注射频率均低于目前的rFⅨ产品[9]。

完成Paradigm 2或Paradigm 3的患者随后被纳入非随机、开放标签扩展试验Paradigm 4，旨在观察N9-GP长期安全性和疗效。结果显示，所有患者均未产生抑制物，不存在与N9-GP相关的安全问题。40 IU/kg预防组中位年化出血次数（ABR）仍维持在1.00（IQR 0～2.03），10 IU/kg预防组中位ABR进一步下降到1.36（IQR 0～2.23），按需止血治疗的成功率为94.6％。14例接受预防治疗的患者接受了23次小手术，围手术期止血效果均被评为“优”或“良”[10]。

Paradigm 5Ⅲ期临床试验对25例≤12岁的患者进行了N9-GP的安全性、疗效和PK评估。患者接受每周1次N9-GP 40 IU/kg的预防治疗，为期52周。结果显示，药物具有良好的安全性、耐受性。N9-GP预防治疗疗效如表3所示，FⅨ平均谷水平可维持在高达0.17 IU/ml，7～12岁年龄组甚至达到0.19 IU/ml，这为N9-GP 40 IU/kg每周1次作为儿童血友病B预防治疗方案提供了有力的依据[11]。

此外，正在进行的Paradigm 6 Ⅲ期临床试验旨在评估<6岁FⅨ活性≤2 IU/dl的未经治疗的患者（PUP）接受每周1次的40 IU/kg预防治疗和每次40～80 IU/kg突破性出血治疗的疗效和安全性。在数据截止日期（2018年8月31日）时，37例患者接受了N9-GP，暴露日（ED）达2833 d，2例因在ED≥10 d时出现高滴度抑制物退出试验。检测到抗N9-GP结合抗体阳性和抗中国仓鼠卵巢（CHO）宿主细胞蛋白抗体阳性患者各5例，未检测到抗PEG抗体，未出现严重药物相关安全性问题。疗效方面，总体中位ABR为0，平均FⅨ谷浓度为0.15 IU/ml。预防组67.9％的患者无出血，15次出血事件只需接受单剂N9-GP治疗后方可止血[12]。

二、重组FⅨ-白蛋白融合蛋白（rⅨ-FP）Albutrepenonacog Alfa

Albutrepenonacog Alfa是首个获批的凝血因子-白蛋白融合的蛋白制品。该药使用基因改造的CHO，通过重组DNA技术将rFⅨ与白蛋白融合，使rFⅨ的半衰期得到延长。同时，通过在rFⅨ与白蛋白之间添加一段可切割序列，保证了FⅨ活性不受影响[13]。该药适用于儿童和成人血友病B患者按需控制和预防出血发作、围术期管理和常规预防治疗。根据欧盟新的产品特性概要（SmPC）中包含的信息，rⅨ-FP是第一个也是唯一一个可用于21 d给药方案的FⅨ产品。rⅨ-FP尚未在中国上市。

rⅨ-FP的临床试验名为“全球PROLONG-9FP临床试验计划”，包括了开放标签、多中心、非随机的Ⅰ期到Ⅲ期研究，评估了rⅨ-FP在中重度（FⅨ活性 ≤2 IU/dl）儿童和成年男性血友病B患者中的疗效和安全性。

rⅨ-FP Ⅰ期剂量递增临床试验旨在评估药物安全性和PK。研究纳入25例12～65岁患者，分别接受剂量为25、50、75 IU/kg的rⅨ-FP。结果显示，患者未发生严重不良事件，没有超敏反应或免疫原性反应的证据。PK分析表明，rⅨ-FP的平均半衰期（92 h）比rFⅨ（17.2 h）和pdFⅨ（14.6 h）延长了5倍以上，回收率增加44％，AUC增加7倍，CL延缓7倍。当rⅨ-FP以每周1次25 IU/kg和每14天1次50 IU/kg给药时，FⅨ谷水平可维持在5％以上，表明每周或隔周给药方案可行[14]。

rⅨ-FP的Ⅲ期研究（3001）旨在评估rⅨ-FP治疗血友病B的PK、疗效和安全性。研究纳入了63例12～65岁经过治疗的患者（PTP），分为预防治疗组（40例）和按需治疗组（23例）。预防组前26周采用每周1次35～50 IU/kg方案，符合要求者随后进入每10天或14天75 IU/kg方案；按需组在治疗26周后转为每周1次预防治疗方案。预防治疗疗效见表2。患者使用rⅨ-FP每周40 IU/kg、每14天75 IU/kg进行预防治疗时，FⅨ谷活性分别为20 IU/dl、12 IU/dl。当受试者从按需治疗转为预防治疗时，中位年化自发出血率（AsBR）下降了100％，中位ABR下降了91％，靶关节消退100％[15]。rⅨ-FP Ⅲ期研究（3002）将目标人群定为0～11岁患者（27例），疗效见表3。rⅨ-FP中位预防剂量为每周46 IU/kg，出血多为创伤造成，97.2％的出血事件可通过1～2次rⅨ-FP注射成功治疗，FⅨ谷活性可维持在13.4 IU/dl[16]。以上两个Ⅲ期研究证明了rⅨ-FP的疗效和安全性：有效控制出血事件和管理围手术期出血；作为预防措施使用可显著降低每年自发性出血的发生率；研究中未发现任何安全性问题，最常见的不良反应是头痛，没有患者出现抑制物、抗CHO或rⅨ-FP抗体。据此，FDA于2016年批准rⅨ-FP上市。

**表2:** 成人血友病B患者接受长效rFⅨ药物预防性治疗临床试验结果

临床试验	给药方案分组	例数	中位ABR（IQR）	中位AsBR（IQR）	半衰期（h）
Collins等（N9-GP）[8]	7 d（10 IU/kg）	30	2.93（0.99～6.02）	0.97（0～4.01）	93.0
	7 d（40 IU/kg）	29	1.04（0～4.00）	0（0～0.98）	
Santagostino等（rⅨ-FP）[15]	7 d	40	0（0～1.87）	0（0～0）	101.7
	10 d	7	0（0～1.78）	0（0～0）	
	14 d	21	1.08（0～2.70）	0（0～1.00）	
Powell等（rFⅨFc）[19]	7 d	63	3.0（1.0～4.4）	1.0（0～2.2）	82.1
	间隔时间调整组	29	1.4（0～3.4）	0.9（0～2.3）	

注：rFⅨ：基因重组凝血因子Ⅸ；rⅨ-FP：rFⅨ-白蛋白融合蛋白；N9-GP：聚乙二醇修饰rFⅨ；rFⅨFc：rFⅨ-Fc融合蛋白；ABR：年化出血次数；AsBR：年化自发出血率；IQR：四分位距

**表3:** 儿童血友病B患者接受长效rFⅨ药物预防性治疗临床试验结果

	给药方案	患者年龄分组	例数	中位ABR（IQR）	中位AsBR（IQR）	半衰期（h）
Carcao等（N9-GP）[11]	7 d（40 IU/kg）	全部患者	25	1.00	0	/
		0～6岁	12	0	0	69.6
		7～12岁	13	2.00	0	76.3
Kenet等（rⅨ-FP）[16]	7 d（35～50 IU/kg）	全部患者	27	3.12（0.91～5.91）	0（0～0.91）	91.4
		0～6岁	12	2.64（2.00～6.48）	0（0～0）	89.6
		6～11岁	15	3.39（0.76～5.91）	0.78（0～1.99）	92.8
Kathelijn等（rFⅨFc）[22]	7 d（50～60 IU/kg）	全部患者	30	2.0（0～3.1）	0（0～1.2）	68.6
		0～6岁	15	1.1（0～2.9）	0（0～1.1）	66.5（11例）
		6～11岁	15	2.1（0～4.2）	0（0～2.1）	70.3（13例）

注：rFⅨ：基因重组凝血因子Ⅸ；rⅨ-FP：rFⅨ-白蛋白融合蛋白；N9-GP：聚乙二醇修饰rFⅨ；rFⅨFc：rFⅨ-Fc融合蛋白；ABR：年化出血次数；AsBR：年化自发出血率；IQR：四分位距；/：无数据

rⅨ-FP Ⅲ期延伸试验（3003）旨在评估近3年rⅨ-FP作为预防和按需治疗的长期安全性和有效性，纳入了完成Ⅲ期3001（52例）、3002（24例）和经历大手术后开始接受预防治疗（7例）的患者。PTP接受rⅨ-FP每周35～50 IU/kg预防方案，符合要求者可更换为每10天或14天50～75 IU/kg方案，并且可以进一步更换为每21天100 IU/kg预防方案。PUP在第1年内给予每周1次rⅨ-FP作为预防和/或按需治疗，随后进行每周常规预防治疗。数据截取至2017年3月，成年患者7、10、14、21 d预防方案的中位AsBR（IQR）分别为0.33（0～2.39）、0（0～0.68）、0.26（0～1.54）、0（0～0.45）。儿童患者7、10、14 d预防方案的中位AsBR（IQR）分别为0（0～0.59）、0（0～3.06）、0.75（0～2.86）。rⅨ-FP中位ED为165 d，77例（92.8％）患者ED>100 d，没有患者产生FⅨ抑制物。该研究证实了rⅨ-FP预防治疗的长期疗效和耐受性良好。rⅨ-FP给药间隔可在特定的成人中延长至21 d，在特定的儿童中延长至10 d、14 d[17]。据此，2020年欧盟批准rFⅨ-FP SmPC更新常规预防治疗方案，支持对部分患者的治疗间隔延长至21 d。

三、重组FⅨ-Fc融合蛋白（rFⅨFc）Eftrenonacog alfa

Eftrenonacog alfa是全球首个人rFⅨ-Fc融合蛋白（rFⅨFc），于2014年获美国FDA批准上市，2021年获中国国家药品监管局批准上市，用于儿童和成人血友病B患者的控制出血、常规预防及围术期管理。该药是目前国内唯一被批准用于按需和常规预防治疗的长效rFⅨ。

Fc融合是一项成熟的延长药物半衰期的技术。rFⅨFc是由IgG1 Fc段共价结合rFⅨ单分子构成。在体内，rFⅨFc从循环中通过非特异性内吞作用进入细胞，在酸性pH环境下与FcRn结合从而避免了被溶酶体降解，延长了药物血浆半衰期。当内体与胞膜融合后，rFⅨFc在中性pH环境下与FcRn分离，被释放回循环系统。rFⅨFc是应用重组DNA技术通过人胚胎肾细胞293经体外培养合成后分离获得，该过程无血源性蛋白的引入，大大降低了病毒污染的风险[3]。基于多项临床研究数据，特别是BLONG、Kids-BLONG为代表的关键Ⅲ期研究数据，rFⅨFc的上市申请于2014被FDA批准。截至目前该药物已在40多个国家及地区上市，大大改善了血友病B患者的治疗现状。下面对rFⅨFc的相关临床研究进行总结和概述。

1. rFⅨFc的Ⅰ/Ⅱa期研究：rFⅨFcⅠ/Ⅱa期开放标签、剂量递增的临床试验旨在评估单次rFⅨFc给药后30 d内的安全性和PK。14例成年PTP（FⅨ活性≤2 IU/dl）完成了试验，分别接受了不同剂量rFⅨFc（剂量分别为1、5、12.5、25、50、100 IU/kg）。结果显示，rFⅨFc活性和FⅨ抗原水平随用药剂量成比例增加，rFⅨFc输注后即刻达到最大浓度，提示rFⅨFc具有类似rFⅨ的快速起效作用。rFⅨFc平均半衰期为56.7 h，约为rFⅨ的3倍。rFⅨFc平均回收率为每IU/kg 0.93 IU/dl，与pdFⅨ相似。FⅨ活性维持在基线1 IU/dl以上的平均时间在25、50、100 IU/kg三个剂量组分别延长了7.34、10.1、12.3 d。安全性方面，患者对rFⅨFc耐受性良好，所有患者均未检出FⅨ抑制物或抗rFⅨFc抗体，大多数不良反应为轻、中度[18]。

2. B-LONGⅢ期研究：rFⅨFc的Ⅲ期开放标签、非随机化的研究（B-LONG）旨在评估rFⅨFc在预防、治疗出血和围术期出血管理的安全性、有效性和PK。研究纳入了123例≥12岁PTP（FⅨ活性≤2 IU/dl），分为4个治疗组：第1组接受每周剂量调整的预防治疗（起始剂量每周50 IU/kg），第2组接受间隔时间调整的预防治疗（起始剂量每10天100 IU/kg），两组调整剂量或间隔使FⅨ活性维持在高于基线1～3 IU/dl；第3组接受按需治疗（20～100 IU/kg），第4组在围术期接受治疗。预防治疗疗效见表2。rFⅨFc单次注射足以解决90.4％的出血事件，第2组中53.8％的患者在研究最后3个月给药间隔≥14 d。第4组手术止血效果评分均为“优”或“良”。所有患者均未检出抑制物，未发生血栓、严重超敏反应事件，这与对血友病B群体的预期一致。PK研究结果显示，rFⅨFc半衰期是rFⅨ的2.43倍，FⅨ活性降至谷浓度1 IU/dl所需时间优于rFⅨ（11.2 d对5.1 d，*P*<0.001）[19]。

此外，Diao等[20]对Ⅰ/Ⅱa期和B-LONG Ⅲ期临床试验进行了事后分析，提出了rFⅨFc群体PK三室模型，为评估和优化血友病B患者给药方案提供了依据。对B-LONG Ⅲ期临床试验手术患者的进一步分析显示，相比于传统FⅨ产品，rFⅨFc消耗量和给药频率更低，可以在围术期更好的维持保护性FⅨ水平和降低出血率。同时，研究首次证实手术对rFⅨFc的PK没有显著影响[21]。

3. 儿童B-LONG Ⅲ期研究（Kids B-LONG）：儿童B-LONG Ⅲ期多中心、开放标签的研究旨在评估rFⅨFc在<12岁PTP（FⅨ活性≤2 IU/dl）中的安全性、有效性和PK。研究方案及结果见表3。没有患者发生rFⅨFc抑制物，未发生药物相关严重不良事件。10例（33％）报告无出血事件，19例（63％）报告无关节内出血事件。rFⅨFc预防治疗中位平均剂量为每周58.6（IQR 52.3～64.8）IU/kg。因此，每周1次rFⅨFc预防治疗对于严重血友病B患儿安全有效，rFⅨFc较长的半衰期有助于减低用药频率，提高患者依从性[22]。

4. B-YOND开放标签扩展研究：B-YOND旨在评估rFⅨFc治疗血友病B长期的安全性和有效性。研究纳入了完成B-LONG（93例）和儿童B-LONG（27例）Ⅲ期研究的患者。受试者接受≥1种治疗方案，包括每周20～100 IU/kg预防治疗、每8～16天1次100 IU/kg的个体化间隔预防、个性化给药以满足个体需求的改良预防治疗或按需治疗，治疗方案可随时切换。其中，来自B-LONG的患者中位治疗时间、中位ED分别为4.0（0.3～5.4）年、146（8～462）d，来自儿童B-LONG的患者分别为2.6（0.2～3.9）年、132（50～256）d。结果显示，药物安全性与先前的研究一致。疗效方面，患者ABR仍保持在低水平，大多数患者（78％）保持了原始研究中的给药间隔。与B-LONG研究相比，B-YOND研究每周中位rFⅨFc消耗量变化不著。研究期间发生了19次可以评估止血疗效的重大手术，疗效均被评为“优”或“良”。其中有16例（76％）需输注1次rFⅨFc以维持止血，中位剂量为94.7（60.6～152.3）IU/kg，手术当天rFⅨFc中位消耗量为136.2（60.6～420.6）IU/kg。术后28 d内，18例（86％）患者未发生出血[23]。

此外，一项B-YOND研究的事后分析评估了具有靶关节的60例患者靶关节状况和生活质量情况。患者rFⅨFc中位用药累积持续时间为3.6（1.4～6.0）年，中位剂量为每周49.7（39.7～64.6）IU/kg，中位给药间隔为7.0（6.9～7.1）d。结果显示，100％靶关节缓解，90.2％的靶关节无复发，中位缓解时长为47.4（19.8～60.6）月，中位AsBR为0（0～0.4）。同时，患者生活质量也显著改善并可维持24个月[24]。

综上，B-YOND研究为rFⅨFc预防、治疗急性出血和围术期管理的长期安全性和有效性提供了证据[23]。

5. PUPs B-LONG研究：PUP是抑制物产生的高危人群，PUPs B-LONG研究是一项多国多中心、开放标签的Ⅲ期临床试验，是首个评估PUP接受rFⅨFc预防及按需治疗的有效性及安全性的研究。研究纳入33例<18岁PUP（FⅨ活性≤2 IU/dl），在开始rFⅨFc预防治疗之前可予按需止血治疗。结果显示，1例（3.0％）在经历11个ED后产生低滴度抑制物（<5.00 BU/ml）。23例（69.7％）发生了58次需紧急治疗的严重不良事件（TESAE），其中2次与治疗相关（1例出现FⅨ抑制物和超敏反应，随后退出试验）。预防治疗中位ABR为1.2，止血治疗所需的rFⅨFc中位注射次数为1。在预防治疗组和止血治疗组中，rFⅨFc疗效被评为“优”、“良”的比例分别为100％、87.7％。因此，PUP接受rFⅨFc预防和止血治疗疗效、耐受性和安全性良好；严重不良事件的类型、发生率与小儿血友病人群的预期相似[25]。

6. 真实世界研究：Shapiro等[26]对美国6个中心接受rFⅨFc预防或按需治疗的64例血友病B患者进行了回顾性分析，患者中位治疗时间为2.7（0.5～5.0）年。预防组中，91％的患者维持或延长了给药间隔时间，每周因子消耗量约减少50％，总ABR、AsBR和年关节出血率在改用rFⅨFc预防后下降。大多数患者对推荐治疗的依从性得到改善或保持稳定。O'Donovan等[27]对成年重型血友病B患者（FⅨ活性≤1 IU/dl）接受两年rFⅨFc预防治疗的结果进行了总结。与rFⅨ相比，rFⅨFc显著改善了中位ABR（3.3对2.0，*P*＝0.01），并减少了28％的因子消耗，每周输注1次rFⅨFc的中位FⅨ谷活性为0.09（0.06～0.14）IU/ml。澳洲的一项包含64例血友病B患者的回顾性研究显示，rFⅨFc注射频率（每周1次）较rFⅨ降低（每周2次），中位ABR降低（2.0对3.0），零出血的患者比例升高（46％对31％），实际因子利用率升高了4 IU/kg/周，研究期间患者无抑制物产生，依从性得到提高[28]。目前正在法国开展的为期两年的真实世界研究（B-SURE）也将会在近期内提供法国血友病B患者使用rFⅨFc疗效与因子使用情况的数据（NCT03655340）。

综上，rFⅨFc的预防治疗可改善血友病B患者的出血发作控制，延长用药间隔，减少用药总量和输注频率，有助于提高血友病B患者的疗效和依从性。此外，rFⅨFc将为患者提供更多个性化的治疗选择。

四、总结及展望

蛋白工程学对rFⅨ进行修饰可能通过延长药物半衰期和提升FⅨ谷水平提高血友病B患者预防治疗的疗效和依从性[29]。多项临床试验结果表明，长效rFⅨ药物在降低ABR的同时，可减少患者注射频率和年因子消耗量，节约医疗资源，减轻患者疾病负担，提高患者生活质量，其用于儿童期预防治疗还可以有效降低致残率，同时药物具有较高的安全性。长效rFⅨ药物的引入有望让中国血友病B患者迎来长效因子预防治疗的模式。
